# Supplementary material for: Copper isotopes track the Neoproterozoic oxidation of cratonic mantle roots
Source: Nat Commun. 2024 May 21;15:4311. doi: 10.1038/s41467-024-48304-2 (PMC11109192; doi:10.1038/s41467-024-48304-2)
Supplement: Supplementary file 1 — Supplementary information [file 41467_2024_48304_MOESM1_ESM.pdf]

Supplementary Materials for

Copper isotopes track the Neoproterozoic oxidation of cratonic mantle roots

Chunfei Chen[1,2*], Stephen F. Foley[1,3], Svyatoslav S. Shcheka[1], Yongsheng Liu[2*]

[1]School of Natural Sciences, Macquarie University, North Ryde, New South Wales 2109, Australia

[2]State Key Laboratory of Geological Processes and Mineral Resources, School of Earth Sciences, China University of Geosciences, Wuhan 430074, China

[3]Research School of Earth Sciences, Australian National University, Canberra, ACT 2601, Australia

*Correspondence to: chfchen2016@hotmail.com and yshliu@hotmail.com

Contents:

Section 1: Supplementary figures (Figures S1-8 used in the main text)

Section 2: Supplementary tables (Tables S1-2 used in the main text)

1. Supplementary figures (Figures S1-8 used in the main text)

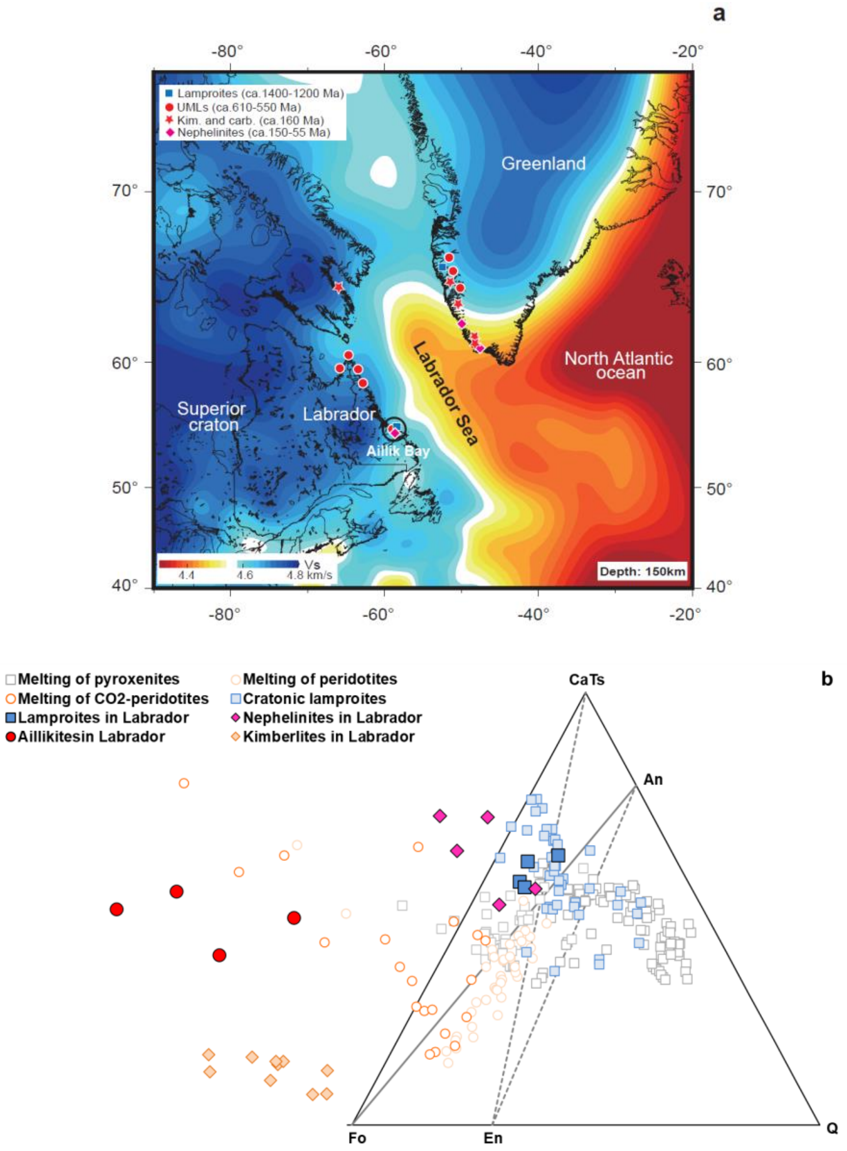

Fig. S1. Geological setting and major elements of the Labrador alkaline rocks. (**a**) The locations of alkaline rocks at the flanks of the rift and seismic shear wave velocities beneath the North Atlantic craton and the Labrador Sea at a depth of 150 km, modified from Chen et al.[1]. The alkaline rocks (UMLs = Ultramafic Lamprophyre; Kim. and carb. = Kimberlites and carbonatites) are distributed along the relatively low-velocity zone at the margins of Greenland and Labrador. (**b**) The alkaline rocks in Labrador plotted on the Ca-Tschermak-Anorthite-Quartz-Enstatite-Forsterite (CaTs-An-Q-En-Fo) plane from Di, after the projection scheme of O'Hara[2]. Experimental melts from the melting of peridotite, pyroxenite, and CO_2-bearing peridotite are shown for comparison (Chen et al.[1] and references therein). Major elements of alkaline rocks in Labrador are from Tappe et al.[3,4] and major elements of kimberlites in Labrador are from Tappe et al.[5]. The cratonic lamproites are from Dharwar Craton[6], Leucite Hills, and Gaussberg[7].

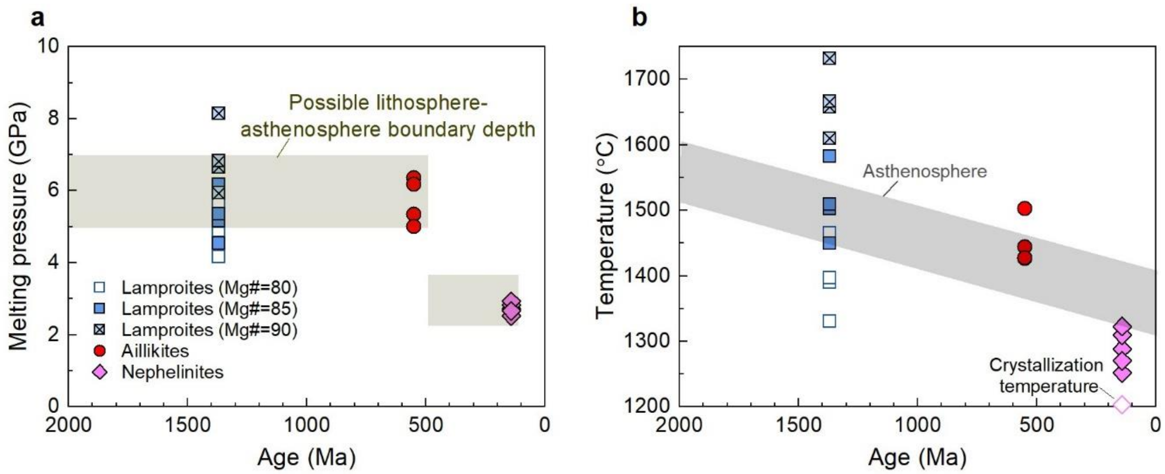

Fig. S2. Melting temperatures and pressures of the Labrador alkaline rocks. Melting pressure (**a**) and temperature (**b**) for the Aillik Bay alkaline rocks were estimated using the method of Ref.[8] (see "Methods"). The olivine Mg# of 80, 85, and 90 in the source of the lamproites were applied to correct primary melt compositions and thus to calculate melting temperatures and pressures[8]. The crystallization temperature of the nephelinite ST103 is shown for comparison. The estimated lithosphere-asthenosphere boundary at Aillik Bay is at 5-7 GPa before 550 Ma and 2-3 GPa between 550 and 140 Ma. The grey region in (**b**) shows the overall range of the potential temperatures of the asthenosphere[9,10]. The results support the role of asthenospheric flow during the formation of the Aillik Bay alkaline rocks at the three stages at the base of the cratonic lithosphere.

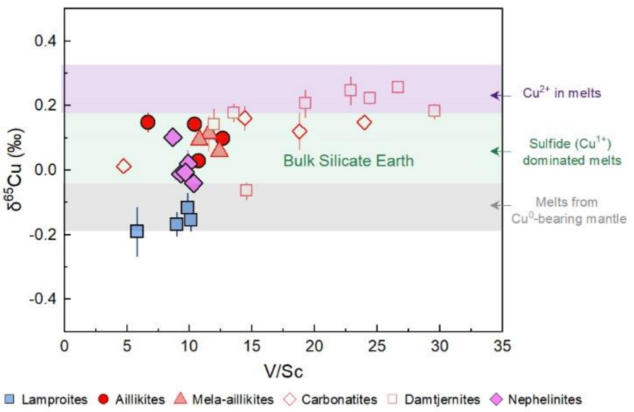

Fig. S3. δ^{65}Cu versus V/Sc of the Labrador alkaline rocks. Copper isotope compositions versus V/Sc ratio of the alkaline rocks from the Labrador in this study. The bulk silicate Earth is shown for comparison[11]. The error bars for all δ^{65}Cu values in this figure are 2sd.

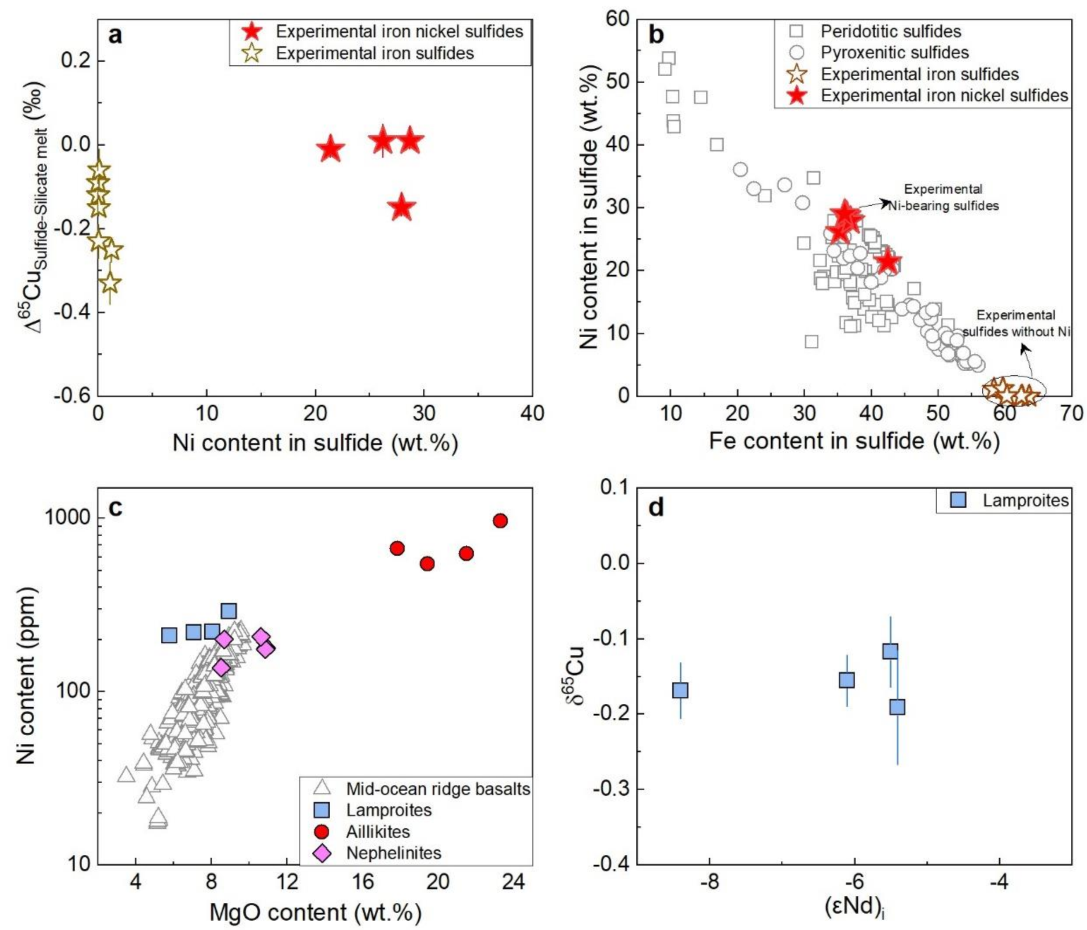

Fig. S4. Copper isotopes versus chemical compositions of sulfides and the Labrador alkaline rocks. (a) Cu isotope fractionation between sulfide and silicate melt versus Ni content in sulfide. (**b**) The compositions of the sulfides in high-pressure experiments[11,12] and in peridotite and pyroxenite xenoliths from global cratons[13-17]. (**c**) MgO versus Ni content in the alkaline rocks from Labrador. The mid-ocean ridge basalts are shown for comparison[18]. (**d**) δ^{65}Cu versus $(\varepsilon Nd)_i$ in the lamproites from Labrador. The error bars for all δ^{65}Cu values in this figure are 2sd.

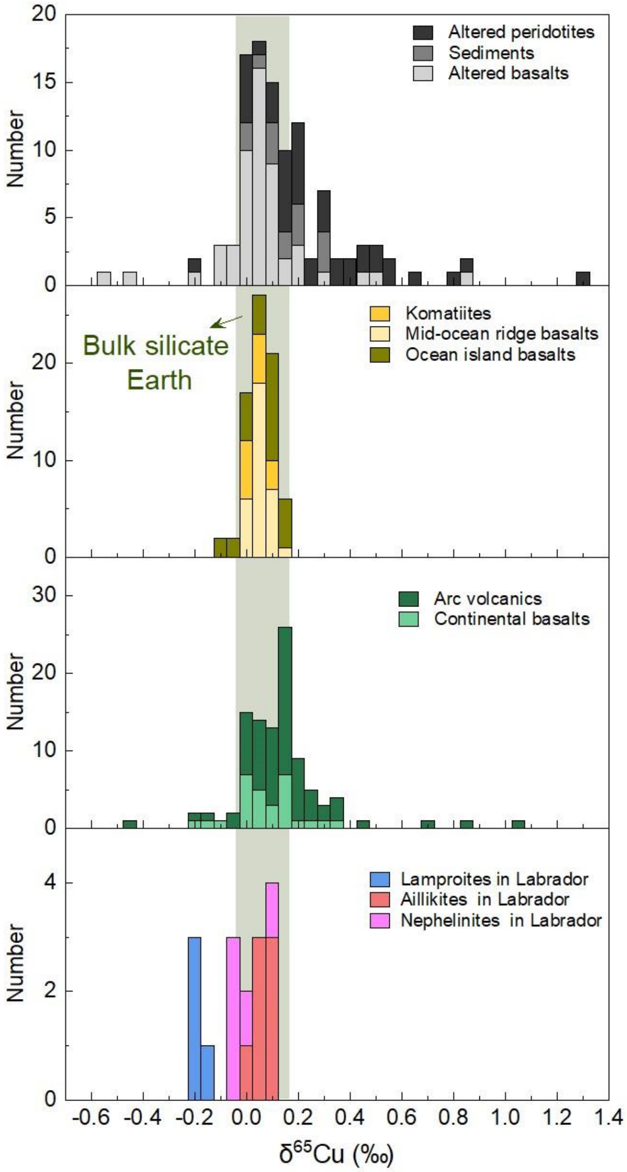

Fig. S5. Compiled δ^{65}Cu of rocks in various geological settings. Compiled Cu isotope compositions of crustal materials (altered oceanic crust[19-21], altered peridotites[22], and sediments[23]), mid-ocean ridge basalts[11,24], komatiites[11], arc volcanics[21,24,25], continental basalts[25], ocean island basalts[11,25], and the alkaline rocks in this study. Bulk silicate Earth is shown for comparison[11].

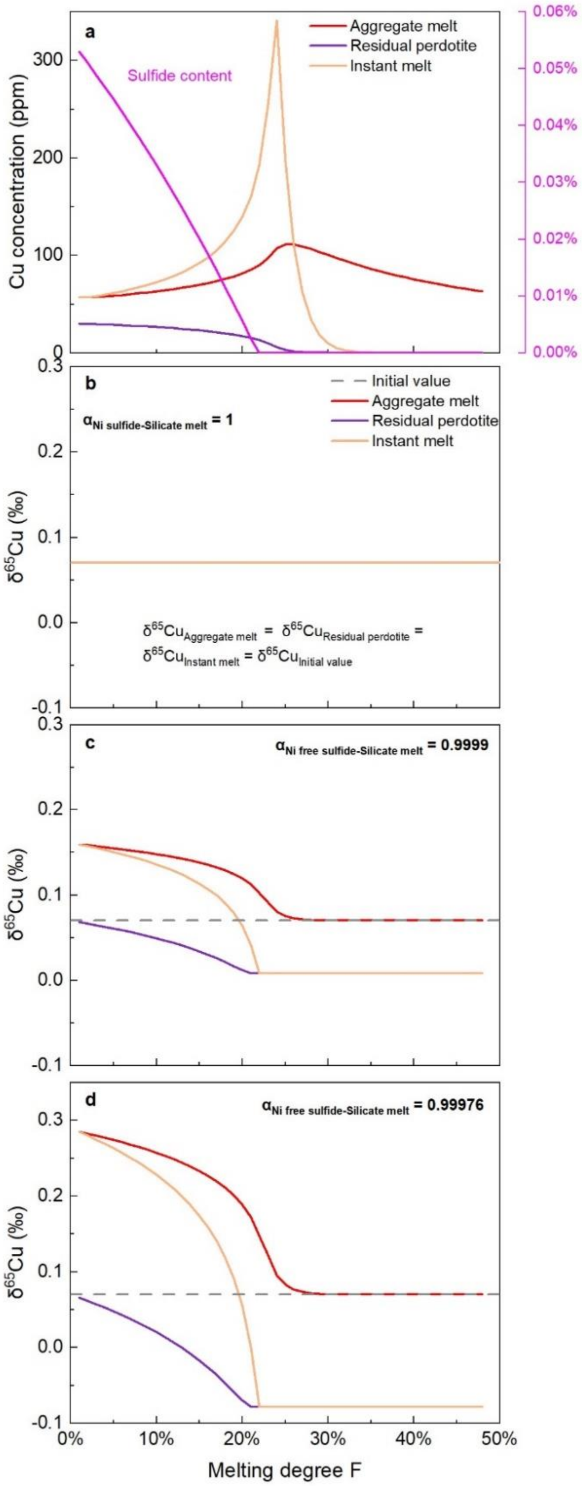

Fig. S6. Modelled Cu isotope fractionation during partial melting of a sulfide peridotite. Modelled Cu content (**a**) and isotope fractionation (**b-d**) during partial melting of a peridotite using Cu isotope fractionation factor $\alpha_{\text{Sulfide-Silicate melt}}$ of 1 (**b**), 0.9999 (**c**), and 0.99976 (**d**). (**a**) Cu contents in melts and residual peridotite and sulfide content in residual peridotite. The δ^{65}Cu in the starting peridotite (initial value) is assumed to be 0.070‰ (Cu isotope composition of the bulk silicate Earth[11]).

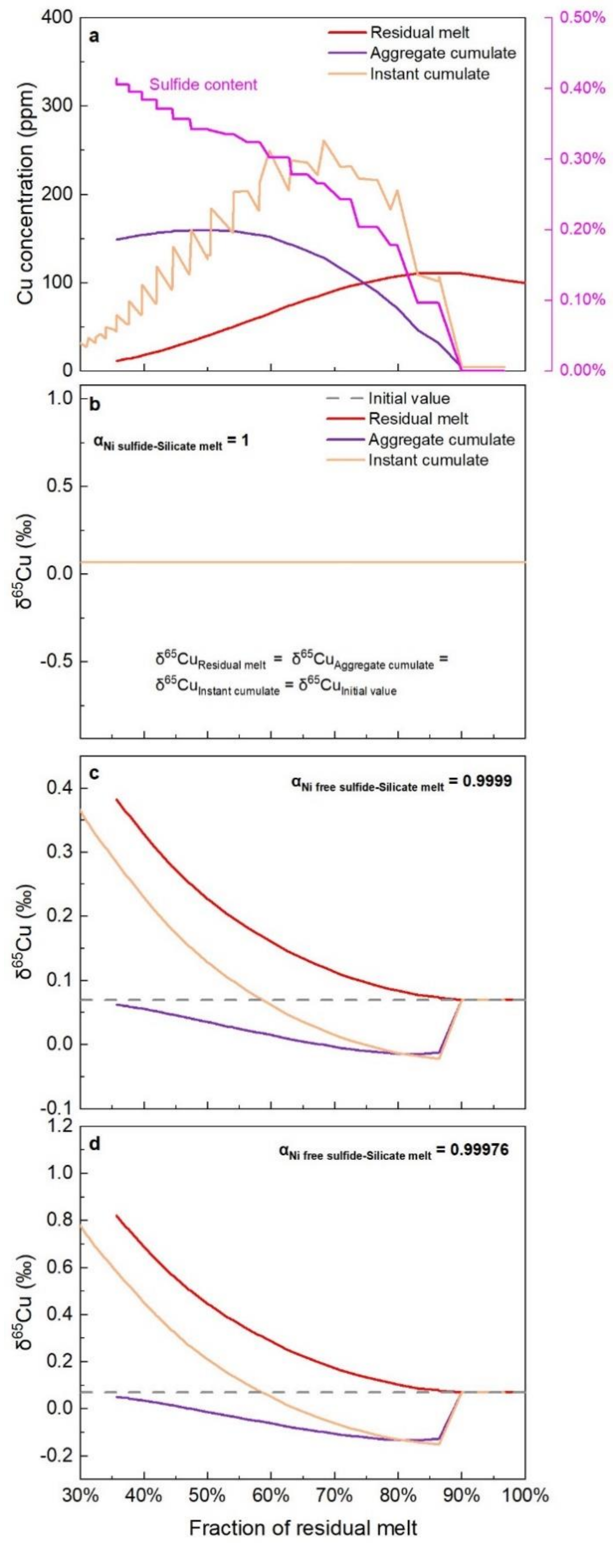

Fig. S7. Modelled Cu isotope fractionation during magmatic differentiation of a basaltic melt. Modelled Cu content (**a**) and isotope fractionation (**b-d**) during magmatic differentiation of a mid-ocean ridge basalt-like melt using Cu isotope fractionation factor $\alpha_{Sulfide\text{-}Silicate\ melt}$ of 1 (**b**), 0.9999 (**c**), and 0.99976 (**d**). The $\delta^{65}Cu$ in the starting peridotite (initial value) is assumed to be 0.070‰ (Cu isotope composition of the bulk silicate Earth[11]).

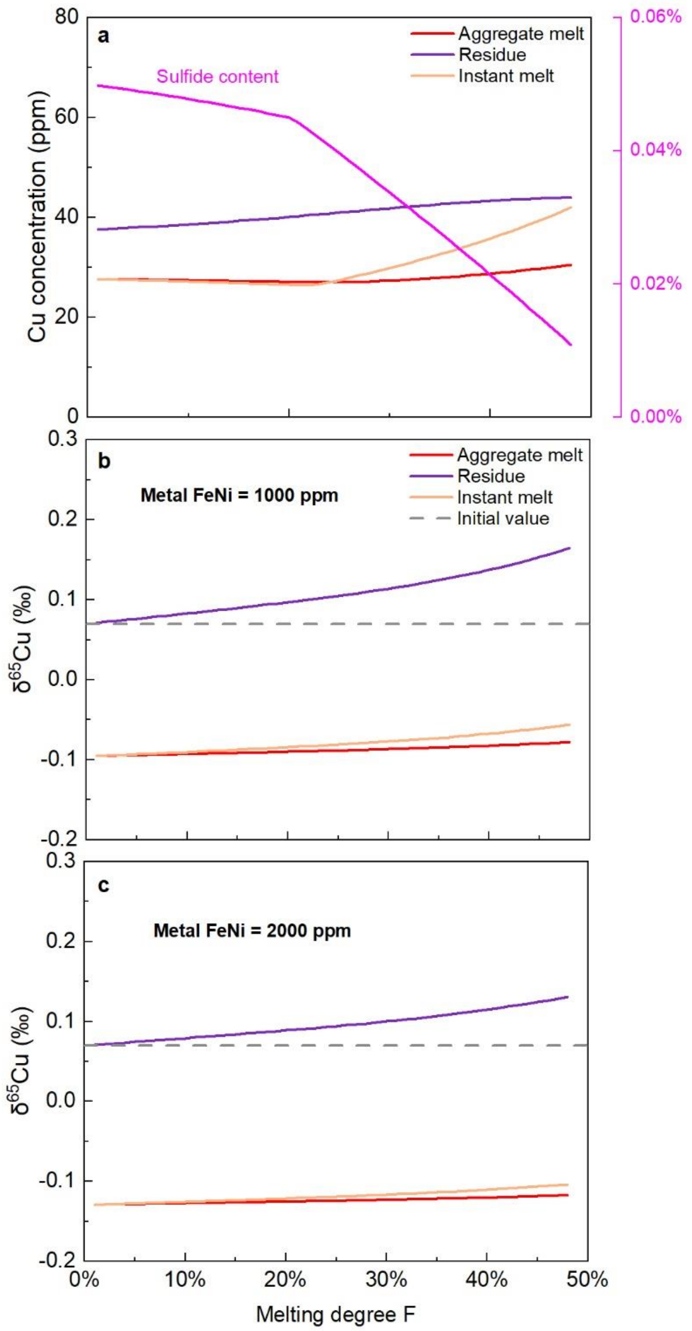

Fig. S8. Modelled Cu isotope fractionation during partial melting of a metal-saturated pyroxenite. Modelled Cu content (**a**) and δ^{65}Cu of silicate melts during partial melting of metal-saturated pyroxenites with 1000 ppm (**b**) and 2000 ppm metal (**c**). The δ^{65}Cu in the starting pyroxenite (initial value) is assumed to be 0.070‰ (Cu isotope composition of the bulk silicate Earth[11]).

2 Supplementary tables (Tables S1-2 used in the main text)

Table S1. Major and trace element compositions and Cu isotopes of the alkaline rocks at the Aillik Bay.

Sample	Rocks	Age	TiO_2	Al_2O_3	FeO	MnO	MgO	CaO	Na_2O	K_2O	P_2O_5	H_2O[a]	C	Sc	V	Cr	Ni	Cu	Zn	Rb	Sr	Y	La	Ce	Pr	Nd	Sm	Eu	Gd	Tb	Dy	Ho	Er	Tm	Yb	Lu	$\delta^{65}Cu$	2sd	n	$\delta^{65}Cu$	2sd
		Ma						wt.%																	ppm												‰			‰	
ST115a	Lamproites	1400	4.1	9.5	11.9	0.14	8.1	7.1	1.9	7.4	1.2	2.0	0.33	16	162	251	220	56	157	141	1229	29	123	263	32	125	20	5.5	13	1.6	7.4	1.1	2.5	0.3	1.6	0.19	-0.16	0.03	3		
ST208	Lamproites	1400	4.0	8.2	11.5	0.13	8.9	6.8	1.7	5.8	0.9	2.0	1.39	16	161	279	290	45	131	104	1584	24	88	191	24	96	16	4.3	11	1.2	5.9	0.9	2.0	0.2	1.2	0.17	-0.12	0.05	3		
ST223	Lamproites	1400	3.6	10.0	10.8	0.16	7.1	9.0	2.5	4.8	0.9	1.3	0.95	19	168	354	218	57	118	94	1121	24	83	182	23	89	14	4.1	10	1.3	5.7	0.9	2.0	0.3	1.5	0.18	-0.17	0.04	3		
ST237	Lamproites	1400	4.9	8.4	12.4	0.15	5.8	6.4	1.6	7.8	1.3	1.7	0.90	17	99	180	209	51	180	161	1920	34	102	237	31	133	23	6.4	16	1.9	8.5	1.2	2.9	0.3	1.7	0.22	-0.19	0.08	3		
ST164	Aillikites	550	3.4	3.5	13.4	0.24	19.4	16.1	0.2	2.4	3.2	2.5	2.81	31	206	758	549	109	114	77	2609	52	247	521	61	237	38	10	27	3.1	14	2.0	4.3	0.5	2.3	0.24	0.15	0.03	3		
ST198a	Aillikites	550	3.9	3.2	13.8	0.21	17.8	12.9	0.6	1.6	1.7	1.7	3.63	19	204	800	672	61	121	43	1434	38	202	450	54	216	34	9.1	23	2.6	11	1.5	3.2	0.4	1.7	0.21	0.03	0.02	3		
ST225	Aillikites	550	2.8	2.3	13.5	0.25	23.3	14.9	0.2	1.3	2.2	2.2	2.75	15	192	422	963	49	132	29	2859	52	283	621	73	285	43	11	28	3.1	14	2.0	4.4	0.5	2.5	0.28	0.10	0.02	3		
ST250a	Aillikites	550	2.7	3.4	12.7	0.25	21.5	15.0	0.2	2.3	1.3	4.7	3.76	19	202	817	626	45	107	80	1456	27	229	492	57	217	30	7.3	19	1.9	8.1	1.1	2.4	0.2	1.2	0.14	0.14	0.02	3		
ST147b	Mela-aillikites	550	6.1	4.3	17.0	0.20	15.3	11.3	0.6	2.3	1.4	3.4	1.25	24	303	688	677	105	158	53	1350	40	123	294	37	155	28	7.5	19	2.4	11.1	1.6	3.4	0.4	1.9	0.23	0.06	0.01	3		
ST196	Mela-aillikites	550	5.0	5.2	15.2	0.19	21.0	9.2	0.5	1.8	0.6	3.9	0.55	26	303	1248	926	117	113	72	593	19	49	109	14	56	11	3.1	8	1.0	4.9	0.7	1.6	0.2	1.1	0.14	0.11	0.05	3		
ST244b	Mela-aillikites	550	5.9	4.0	15.1	0.19	22.5	10.3	0.6	1.7	1.0	3.0	1.47	26	284	1144	931	83	127	51	991	29	120	272	34	138	25	6.7	16	2.0	8.6	1.2	2.5	0.3	1.3	0.15	0.09	0.01	3		
ST140	Damtjernites	550	3.8	7.4	12.5	0.30	7.6	17.1	1.0	2.3	2.3	2.5	0.52	13	389	11	32	145	246	36	1478	82	191	301	31	120	26	8.0	25	3.5	20	3.1	7.3	0.9	4.6	0.56	0.18	0.02	3		
ST174	Damtjernites	550	6.3	8.5	15.8	0.34	6.1	17.2	2.2	2.5	2.4	3.6	0.05	17	392	4.9	11	66	208	56	3596	105	386	846	100	388	70	20	50	6.5	31	4.6	9.8	1.1	5.1	0.62	0.25	0.04	3		
ST188a	Damtjernites	550	4.9	9.0	14.8	0.32	6.8	16.8	5.5	1.0	2.8	2.9	0.63	14	338	4.8	31	91	135	37	2080	97	332	619	67	251	48	14	38	5.1	25	3.8	8.6	1.0	5.5	0.70	0.22	0.01	3		
ST206a	Damtjernites	550	7.2	7.2	15.8	0.22	8.1	17.1	1.0	2.2	2.9	1.8	1.31	26	384	26	98	147	176	57	1428	89	300	688	83	332	64	18	48	6.0	27	3.8	7.5	0.8	3.9	0.43	-0.06	0.03	3	0.00	0.01
ST224b	Damtjernites	550	5.7	7.5	13.4	0.24	6.4	16.8	1.5	2.4	3.4	1.8	1.99	31	368	57	38	124	150	66	2529	76	223	500	63	262	48	14	35	4.4	20	3.0	6.6	0.8	3.8	0.45	0.14	0.05	3		
ST226	Damtjernites	550	6.0	10.2	14.9	0.35	6.6	13.7	2.0	2.3	1.8	3.0	0.71	13	354	56	68	78	205	60	1407	89	312	644	75	291	54	15	39	5.1	23	3.5	7.7	0.9	4.7	0.58	0.26	0.02	3		
ST114	Damtjernites	550	5.4	3.8	15.7	0.23	19.7	11.7	0.3	2.2	1.6	1.1	2.21	21	280	961	831	86	139	52	2152	41	165	383	47	190	31	8.1	21	2.5	11	1.6	3.6	0.4	2.0	0.24	0.18	0.03	3		
ST170	Damtjernites	550	5.3	9.5	13.0	0.22	7.1	15.3	1.2	2.2	1.6	2.5	0.30	16	315	7.9	26	68	155	29	1111	80	256	610	75	295	52	14	36	4.6	21	3.2	7.2	0.8	4.2	0.49	0.21	0.04	3		
ST126	Carbonatites	550	2.5	2.3	11.2	0.28	11.8	22.5	0.2	1.5	0.5	2.7	7.09	28	132	684	240	25	161	85	1213	49	282	619	70	249	30	6.0	17	1.8	8.9	1.6	5.5	1.1	9.8	1.70	0.01	0.01	3		
ST193a	Carbonatites	550	1.4	2.0	6.6	0.41	11.0	29.5	0.2	1.3	1.4	1.6	8.32	13	248	238	222	55	108	45	2811	39	403	610	62	220	30	7.2	19	2.0	8.7	1.3	3.1	0.4	2.3	0.28	0.12	0.06	3		
ST198c	Carbonatites	550	2.4	1.8	9.4	0.31	7.3	29.6	1.2	0.7	5.0	0.6	7.01	13	191	14	27	46	147	21	6375	140	598	1340	165	647	107	27	70	8.0	35	5.2	11.5	1.3	7.0	0.82	0.16	0.04	3		
ST199	Carbonatites	550	1.9	2.0	8.5	0.31	8.1	26.4	2.0	1.5	3.8	1.5	6.60	15	357	66	86	56	116	42	3260	65	356	677	77	296	47	12	32	3.7	16	2.5	5.4	0.6	3.3	0.40	0.15	0.01	3		
ST100	Nephelinite	140	2.6	12.3	11.0	0.17	10.9	13.7	0.8	1.8	1.5	5.5	0.29	27	253	287	179	75	82	52	902	31	103	223	26	103	17	4.7	12	1.6	7.3	1.2	2.9	0.3	2.1	0.28	-0.01	0.02	3		
ST102	Nephelinite	140	1.8	14.2	11.1	0.22	8.5	9.8	3.1	2.2	0.8	3.9	0.17	23	197	247	138	55	138	54	1124	34	104	206	23	85	14	3.6	10	1.3	6.5	1.2	3.0	0.4	2.6	0.34	0.10	0.01	3		
ST103	Nephelinite	140	2.2	10.8	11.4	0.29	10.8	12.5	2.7	1.8	2.6	6.4	1.01	23	235	220	176	67	99	48	1792	45	218	438	49	187	29	7.5	20	2.3	11	1.7	4.0	0.5	2.8	0.36	-0.04	0.02	3	-0.05	0.00
ST253	Nephelinite	140	2.6	12.4	12.3	0.22	8.7	12.8	3.0	1.9	1.0	1.7	0.98	26	254	308	201	65	105	52	1262	30	91	187	22	86	15	4.1	11	1.4	6.8	1.1	2.7	0.4	2.1	0.26	0.02	0.04	3		
ST254	Nephelinite	140	2.3	12.0	11.8	0.21	10.6	14.1	3.3	1.7	1.2	1.6	1.02	26	256	273	208	63	93	37	1636	32	126	245	28	104	17	4.5	12	1.5	7.0	1.2	2.9	0.3	2.2	0.27	-0.01	0.01	3		

a. H_2O and CO_2 contents are from Ref.[3] and Ref.[4]. b. Replicate analysis of the same sample powder.

Tables S2. Copper isotopes compositions of USGS standards.

Samples	References	Cu (ppm)	δ^{65}Cu (‰)	2sd	n
BHVO-2	This study	135	0.11	0.02	3
	Zhu et al. (2019)[26]		0.14^{a}	0.03	47
	Moynier et al. (2017)[27]		0.12^{b}	0.05	
	Savage et al. (2015)[11]		0.10	0.08	3
	Liu et al. (2014)[28]		0.15	0.05	18
BIR-1	This study	125	0.05	0.01	3
	Zhu et al. (2019)[26]		0.01	0.03	17
	Moynier et al. (2017) [27]		0.02	0.11	
	Savage et al. (2015) [11]		0.09	0.01	2
	Liu et al. (2014)[28]		0.00	0.05	6

a: The results of the long-term measurement on Cu isotopes of the standards in the lab which is used in this study.

b: The recommended values from Moynier et al. (2017) are summarized from previously published data.

References

1 Chen, C. *et al.* Calcium isotopes track volatile components in mantle sources of alkaline rocks and associated carbonatites. *Earth and Planetary Science Letters* **625**, 118489 (2024).

2 O'Hara, M. J. The bearing of phase equilibria studies in synthetic and natural systems on the origin and evolution of basic and ultrabasic rocks. *Earth-Science Reviews* **4**, 69-133 (1968).

3 Tappe, S. *et al.* Genesis of Ultramafic Lamprophyres and Carbonatites at Aillik Bay, Labrador: a Consequence of Incipient Lithospheric Thinning beneath the North Atlantic Craton. *Journal of Petrology* **47**, 1261-1315 (2006).

4 Tappe, S. *et al.* Craton reactivation on the Labrador Sea margins: 40Ar/39Ar age and Sr–Nd–Hf–Pb isotope constraints from alkaline and carbonatite intrusives. *Earth and Planetary Science Letters* **256**, 433-454 (2007).

5 Tappe, S. *et al.* A fresh isotopic look at Greenland kimberlites: Cratonic mantle lithosphere imprint on deep source signal. *Earth and Planetary Science Letters* **305**, 235-248 (2011).

6 Talukdar, D. *et al.* Petrology and geochemistry of the Mesoproterozoic Vattikod lamproites, Eastern Dharwar Craton, southern India: evidence for multiple enrichment of sub-continental lithospheric mantle and links with amalgamation and break-up of the Columbia supercontinent. *Contributions to Mineralogy and Petrology* **173**, 67 (2018).

7 Ngwenya, N. S. & Tappe, S. Diamondiferous lamproites of the Luangwa Rift in central Africa and links to remobilized cratonic lithosphere. *Chemical Geology* **568**, 120019 (2021).

8 Sun, C. & Dasgupta, R. Thermobarometry of CO2-rich, silica-undersaturated melts constrains cratonic lithosphere thinning through time in areas of kimberlitic magmatism. *Earth and Planetary Science Letters* **550**, 116549 (2020).

9 Herzberg, C., Condie, K. & Korenaga, J. Thermal history of the Earth and its petrological expression. *Earth and Planetary Science Letters* **292**, 79-88 (2010).

10 Lee, C.-T. A., Luffi, P., Plank, T., Dalton, H. & Leeman, W. P. Constraints on the depths and temperatures of basaltic magma generation on Earth and other terrestrial planets using new thermobarometers for mafic magmas. *Earth and Planetary Science Letters* **279**, 20-33 (2009).

11 Savage, P. S. *et al.* Copper isotope evidence for large-scale sulphide fractionation during Earth's differentiation. *Geochemical Perspectives Letters* **1**, 53-64 (2015).

12 Xia, Y., Kiseeva, E., Wade, J. & Huang, F. The effect of core segregation on the Cu and Zn isotope composition of the silicate Moon. *Geochemical Perspectives Letters* **12** (2019).

13 Westerlund, K. J. *et al.* A subduction wedge origin for Paleoarchean peridotitic diamonds and harzburgites from the Panda kimberlite, Slave craton: evidence from Re–Os isotope systematics. *Contributions to Mineralogy and Petrology* **152**, 275

(2006).
14 Richardson, S. H., Shirey, S. B., Harris, J. W. & Carlson, R. W. Archean subduction recorded by Re–Os isotopes in eclogitic sulfide inclusions in Kimberley diamonds. *Earth and Planetary Science Letters* **191**, 257-266 (2001).
15 Pearson, D. G., Shirey, S. B., Harris, J. W. & Carlson, R. W. Sulphide inclusions in diamonds from the Koffiefontein kimberlite, S Africa: constraints on diamond ages and mantle Re–Os systematics. *Earth and Planetary Science Letters* **160**, 311-326 (1998).
16 Aulbach, S. *et al.* Mantle formation and evolution, Slave Craton: constraints from HSE abundances and Re–Os isotope systematics of sulfide inclusions in mantle xenocrysts. *Chemical Geology* **208**, 61-88 (2004).
17 Aulbach, S. *et al.* Sulfide and whole rock Re–Os systematics of eclogite and pyroxenite xenoliths from the Slave Craton, Canada. *Earth and Planetary Science Letters* **283**, 48-58 (2009).
18 Jenner, F. E. & O'Neill, H. S. C. Analysis of 60 elements in 616 ocean floor basaltic glasses. *Geochem. Geophys. Geosyst.* **13** (2012).
19 Huang, J., Liu, S.-A., Wörner, G., Yu, H. & Xiao, Y. Copper isotope behavior during extreme magma differentiation and degassing: a case study on Laacher See phonolite tephra (East Eifel, Germany). *Contributions to Mineralogy and Petrology* **171**, 76 (2016).
20 Dekov, V. M., Rouxel, O., Asael, D., Hålenius, U. & Munnik, F. Native Cu from the oceanic crust: Isotopic insights into native metal origin. *Chemical Geology* **359**, 136-149 (2013).
21 Wang, Z. *et al.* Evolution of copper isotopes in arc systems: Insights from lavas and molten sulfur in Niuatahi volcano, Tonga rear arc. *Geochimica et Cosmochimica Acta* **250**, 18-33 (2019).
22 Liu, S. A., Liu, P. P., Lv, Y., Wang, Z. Z. & Dai, J.-G. Cu and Zn isotope fractionation during oceanic alteration: Implications for Oceanic Cu and Zn cycles. *Geochimica et Cosmochimica Acta* **257**, 191-205 (2019).
23 Little, S. H., Vance, D., McManus, J., Severmann, S. & Lyons, T. W. Copper isotope signatures in modern marine sediments. *Geochimica et Cosmochimica Acta* **212**, 253-273 (2017).
24 Wang, Z. *et al.* Copper recycling and redox evolution through progressive stages of oceanic subduction: Insights from the Izu-Bonin-Mariana forearc. *Earth and Planetary Science Letters* **574**, 117178 (2021).
25 Liu, S.-A. *et al.* Copper isotopic composition of the silicate Earth. *Earth and Planetary Science Letters* **427**, 95-103 (2015).
26 Zhu, Y. *et al.* High-precision Copper and Zinc Isotopic Measurements in Igneous Rock Standards Using Large-geometry MC-ICP-MS. *Atomic Spectroscopy* **40**, 6 (2019).
27 Moynier, F., Vance, D., Fujii, T. & Savage, P. The Isotope Geochemistry of Zinc and Copper. *Reviews in Mineralogy and Geochemistry* **82**, 543-600 (2017).
28 Liu, S.-A. *et al.* High-precision copper and iron isotope analysis of igneous rock standards by MC-ICP-MS. *Journal of Analytical Atomic Spectrometry* **29**, 122-133 (2014).
