# Supplementary material for: Copper isotopes track the Neoproterozoic oxidation of cratonic mantle roots
Source: Nat Commun. 2024 May 21;15:4311. doi: 10.1038/s41467-024-48304-2 (PMC11109192; doi:10.1038/s41467-024-48304-2)
Supplement: Supplementary file 3 — Description of Additional Supplementary Files [file 41467_2024_48304_MOESM3_ESM.pdf]

Description of Additional Supplementary Files

Supplementary Data 1: Compilation of age and geologic setting of global nonorogenic lamproites.
Supplementary Data 2: Modelling Cu isotope fractionation during partial melting of peridotite at 1.5 GPa.

Supplementary Data 3. Modelling Cu isotope fractionation during magma fractionation of a MORB melt at 0.2 GPa.

Supplementary Data 4: Modeling Cu isotope fractionation during partial melting of a metalsaturated pyroxenite at 6 GPa.
